# Supplementary material for: k-mer manifold approximation and projection for visualizing DNA sequences
Source: Genome Res. 2025 May;35(5):1234–46. doi: 10.1101/gr.279458.124 (PMC12047656; doi:10.1101/gr.279458.124)
Supplement: Supplement 8 [file Supplemental_Data_1.zip › Supplemental_Data_1/ZNF713_TAGGAT20NAGT_AD_3/ZNF713_TAGGAT20NAGT_AD_3_tSNE.pdf]

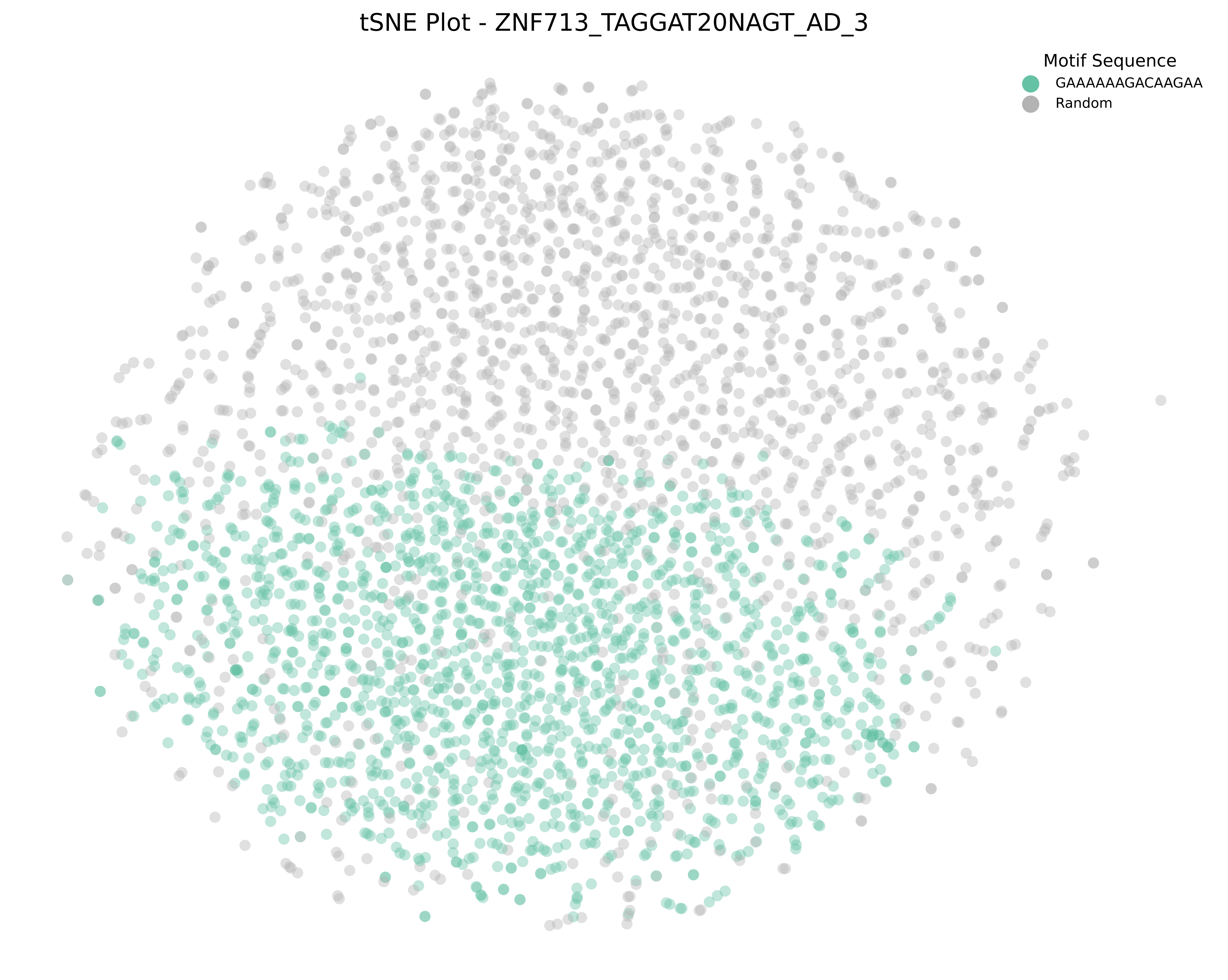
tSNE Plot - ZNF713_TAGGAT20NAGT_AD_3
Motif Sequence
GAAAAAAGACAAGAA
Random
